# Oral health matters in cognitive impaired aged residents in geriatric care facilities: A cross‐sectional survey

**DOI:** 10.1002/nop2.683

**Published:** 2020-11-22

**Authors:** Lan Chen, Liyan Gu, Xianchen Li, Wenyao Chen, Lingjuan Zhang

**Affiliations:** ^1^ Education and Scientific Research Department of Clinical Nursing Changhai Hospital affiliated to Naval Medical University Shanghai China; ^2^ Education and Scientific Research Department of Clinical Nursing NO. 905 Hospital of the Navy Shanghai China; ^3^ Clinical Research Center Shanghai General Hospita Shanghai Jiao Tong University School of Medicine Shanghai China; ^4^ Shanghai Quality Control Center of Geriatric Care Shanghai China

**Keywords:** aged residents, cognitive impairment, geriatric care facilities, OHAT, oral health

## Abstract

**Aims:**

To investigate the oral health status of aged residents and explore the possible factors associated with oral health and the effect of cognitive impairment (CI) on it in geriatric care facilities (GCF) using oral health assessment tool (OHAT) in Shanghai, China.

**Background:**

Oral health is closely associated with overall health. Problems like missing teeth, dental caries, dental pain, periodontal diseases, oral infections and dysphagia are common in GCFs. Furthermore, residents in GCFs with CI are becoming a dominant group and this prevalence increases with age. Detection of oral problems earlier and taking oral care actions is required for these special populations.

**Methods:**

The study was an observational cross‐sectional study conducted in 42 GCFs. Data were collected from 657 subjects, including oral health assessment (OHAT), cognitive impairment (Mini‐Mental State Examination, MMSE) and respondents’ characteristics. The subjects were divided into CI group and non‐CI group based on MMSE. Oral health conditions were compared between the two groups.

**Results:**

Oral health status in the CI group was significantly worse than that in the non‐CI group (*p* < .001) with four OHAT dimensions (tongue (*p* = .0007), saliva (*p* = .0011), natural teeth (*p* = .0155) and oral cleanliness (*p* < .001)). The worst dimension was natural teeth. Debris and plaque index (*p* < .001), oral odour (*p* < .001), chewing function (*p* = .0151) and swallowing function (*p* = .0405) were worse in CI group than those in non‐CI group. In the CI group, providing oral care was a protective factor in oral health (OR = 0.600 95CI% (0.39–0.92)) and wearing dentures was a risk factor (OR = 2.09, 95CI% (1.31–3.32)), while the similar effects were not found in non‐CI group.

**Conclusions:**

Oral health status among aged residents in GCFs in China was worse among individuals with CI. Caregivers in GCFs should focus more on seniors’ oral health with CI.

**Relevance to Clinical Practice:**

Residents who are suffering from CI are more vulnerable to have oral problems. Regular and proper oral health check‐ups in daily nursing work to define oral problems of residents are significant. Nursing staff should pay more attention to oral assessment and effective intervention.

## 1 INTRODUCTION

With the acceleration of society ageing process, the risk of oral‐related disease increases, especially in institutionalized elders(Wong et al., 2019). Oral health issues such as missing teeth, dental caries, dental pain, periodontal diseases, oral infections and dysphagia are common in geriatric care facilities (GCFs) (Chalmers & Pearson, 2005; Wong et al., 2019). This is concerning because oral health is closely associated with overall health. An undesirable set of teeth or deteriorating oral health may lead to malnutrition, endocarditis and aspiration pneumonia (Razak et al., 2014).

Oral hygiene and oral healthcare of residents in GCFs are reported to be generally insufficient around the world. These seniors commonly have complicated oral problems, including large amounts of plaques, debris and calculus and even moderate to advanced periodontitis (Zenthofer et al., 2014). It was reported 78.9% of the surveyed residents in Germany had moderate or deep periodontal pockets (Ziebolz et al., 2017) and visible plaque was found in all residents in Victorian nursing homes and more than 25% of individuals had plaque covering over one‐third of at least one tooth (Hopcraft et al., 2012a). A research in North Carolina discovered that denture hygiene was poor either, especially the upper dentures (especially unexposed surfaces) (Zimmerman et al., 2017). Meanwhile, oral care is not in compliance with the existing international evidence‐based best practice guidelines and protocols; therefore, the residents do not regularly receive the best practice. A study in Canada found that 59% of care providers felt rushed in their last shift when providing oral care to residents and 19% did not complete oral care (Knopp‐Sihota et al., 2015).

In GCFs, residents with dementia or cognitive impairment (CI) are becoming a dominant group and this prevalence increases with age. CI is a status of cognitive decline, the gradual loss of one's ability to learn, remember, pay attention and make decisions. Oral problems are more compromised in these residents and oral hygiene is unacceptable (Ziebolz et al., 2017) and may be at higher risk of developing oral diseases. Providing oral care is further complicated by cognitive or behavioural impairments, resistance and lack of cooperation, from a report of Australia research (Chalmers & Pearson, 2005); low priority of dental services among geriatric residents, poor support for caregivers and lack of staff, time, knowledge, GCF protocols, and regulations (Chalmers & Pearson, 2005; Hoben et al., 2017). This is an alarming phenomenon worldwide documented in numbers of studies reporting poor care and outcomes. Some exploratory mouth‐care projects have been developed; however, the effects are rarely obvious and often temporary. Proper oral health among the institutionalized aged residents with CI around the world is not yet secured. As a result, improving oral health of these population becomes a vital topic for future studies.

In China, the similar dilemma exists. As the population ages, more elderly Chinese individuals are living in GCFs with declining cognitive function, exhibiting various types and degrees of CI, accompanied by chronic and aggressive mental and behavioural abnormalities, all of which complicate daily care(Chan et al., 2013). However, studies on the oral health of this specific group of GCF individuals were not reported and individuals with dementia or CI in GCFs have always been excluded from researches.

The purpose of this study was to explore the oral health situation of aged residents and the effects of CI on oral health. Results are useful not only to the nurses who working in geriatric fields, but also to interdisciplinary dental audience, for guiding future quality improvement efforts.

## 2 MATERIALS AND METHODS

### 2.1 Participants

We calculated a sample size of 531 to allow for stratification by GCFs depends on an α of 0.05, a relative error of sampling of 3% and non‐response rate of 10% with assuming a prevalence of server oral health issues of 20%. A proportional sampling method (10%) with stratification factor of GCF was used to obtain a representative sample of aged residents in all of 42 GCFs in shanghai. A total of 700 residents were obtained by using computer‐generated random numbers. The inclusion criteria were over 60 years and stayed in GCF at least one month. The exclusion criteria were having any life‐threatening, serious medical problems, inability to undergo assessment or cooperate. And 43 subjects were excluded because of having any of above situations. Finally, 657 subjects participated in oral health status assessments and completed the survey for the study.

### 2.2 GCF characteristics

Corresponding information on GCFs was obtained from administrators based on face‐to‐face interviews, including facility size, private or public ownership, established date, doctor–patient ratio, nurse–patient ratio, nurse assistant–patient ratio and GCF’s policy on regular oral health assessment.

### 2.3 Demographical characteristics

Sex, age, marital status, BMI, concomitant disease, cognitive impairment, Barthel Index, length of GCF‐stay, payment with medical insurance and regular family visits were collected.

### 2.4 Assessment of cognitive impairment

Individuals were evaluated for cognitive status using the Mini‐Mental State Examination (MMSE) (Folstein et al., 1975). Participants were asked to solve 30 tasks in six dimensions: orientation, registration, attention, calculation, recall and language. Correctly executed exercises were awarded 1 point, with a total score ranging from 0 to 30, MMSE scores of <24 are indicative of cognitive impairment or dementia. This scoring is considered the gold standard for dementia screening.

### 2.5 Oral characteristics

Trained nursing staff performed the following non‐invasive dental examinations and measurements with a headlight in the participant rooms. Double checks were performed to ensure the integrity and accuracy of the information. Residents were examined using OHAT, it is a simple indicator of overall oral health validated by Chalmers et al. in 2005 (Chalmers et al., 2005) and is widely translated to different versions and applied. It consists of eight items: lips, tongue, gum and tissues, saliva, natural teeth, dentures, oral cleanliness and dental pain. This tool is not requiring patients to express themselves, so persons with cognitive disorders could also be evaluated. Scores on each item range from 0 (healthy) to 2 (unhealthy). The total score ranges from 0 to 16, can be classified into three categories: “healthy mouth” (0–3); “changing mouth” (4–8); “unhealthy mouth” (9–16). The oral environment refers to plaque index and oral odour. Plaque index was measured visually using a probe to examine for soft accumulations, with scores ranging from 0 to 3 (Oltdrn, 1972). Oral odour was also examined by instructing participants to open their mouths and make an "ah~" sound for 5 s; the odour was then judged and recorded in five degrees. Oral function scoring included determination of chewing function in 3 degrees and swallowing function in 5 degrees. Information on oral health behaviours, including brushing, rinsing, oral care procedure, was collected via interview and observation.

### 2.6 Data collection

Data collection was conducted from February to October in 2019. In each GCF, all the research staff were trained and calibrated. Participant's cognitive evaluation was completed by trained nurse first and then the nurse continued to finish the oral assessment. Double checks between two trained nurses were performed to ensure the integrity and accuracy of the information. Data collection for this article was conducted from February to October in 2019.

### 2.7 Ethical considerations

This study was approved by the ethics committee. Written informed consent was signed by all participants or their legally authorized representatives if resident was unable to. We also considered the freedom to withdraw consent during the process, the absence of any detriment in doing so and the anonymity of data.

### 2.8 Statistical analysis

Continuous variables were expressed as mean with standard deviation or median with 25 percentile and 75 percentile and compared using *t* test or Wilcoxon signed‐rank test, as per the normality of data. Categorical variables were expressed as *N* (%) and compared using chi‐square or Fisher's exact tests. For oral health and oral health‐related behaviours, CI group was compared with non‐CI group based on MMSE, respectively. A multivariate logistic regression analysis was performed to identify the possible associations between factors and oral health status. Sex, age, cognitive impairment, education level, regular family visit, institution ownership, providing oral care or not, denture use, length of time to wear dentures, chewing function and swallowing function were entered in the model and selected by a method of stepwise. In the multivariate analysis, we also considered the potential interactions among variables. Values of *p* < .05 were considered significant. All statistical analyses were performed by SAS 9.4(SAS, Cary, NC).

## 3 RESULTS

### 3.1 Participants and GCF characteristics

A total of 657 residents received oral health assessments. Sample characteristics are shown in Table 1. The mean age of the participants was 85.65 years. Participants were primarily female 372 (56.6%) and married 627 (95.4%) and 631 (96.0%) payment with medical insurance. MMSE assessments showed that 412 (62.7%) of the aged residents had cognitive impairment, whereas only 245 (37.3%) had normal cognition. GCFs (*N* = 42) covered 32 (76.2%) private ones and 10 (23.8%) public ones. A total of 475 (72.3%) of the 657 residents lived in private GCFs and others were in public GCFs. The number of beds per GCF varied from the smallest at 50 to the largest at 1,780 beds (private). In terms of total human resource allocation, each doctor cared for 20 people, each nurse cared for 7.69 people and each nurse assistant cared for 5.26 people (shifts are not taken into account).

**TABLE 1:** Characteristics of Respondents

	Median (P25, P75)/*N*(%)
Residents characteristics (*N* = 657)	
Age	85.65 (78.09–89.87)
Sex	
Male	285 (43.4)
Female	372 (56.6)
Marriage (Yes)	627 (95.4)
Length of stay (Days)	94.00 (40.00,231.00)
Payment with medical insurance (Yes)	631 (96.0)
Family regular visits (Yes)	483 (73.5)
Concomitant disease	
Hypertension	414 (63.0)
Diabetes	201 (30.6)
Coronary Disease	259 (29.4)
Tumour	9 (1.4)
Respiratory disease	75 (11.4)
Digestive disease	30 (4.6)
Cognitive impairment	412 (62.7)
Institution characteristics (*N* = 42)	
For‐profit ownership	
Private	33 (76.7)
Public	10 (23.3)
Nursing home established time (Years)	6.0 (4.00,17.00)
Number of beds	200 (110.00,270.00)
Human resource ratio	
Ratio doctor–patient	0.05 (0.03,0.06)
Ratio nurse–patient	0.13 (0.11,0.17)
Ratio nurse assistant–patient	0.19 (0.18,0.25)

### 3.2 Measuring oral health status using OHAT

The overall distribution of OHAT scores in two groups is presented in Table 2. Overall, oral health status in the CI group was significantly worsen than that in the non‐CI group (*p* < .001), only 281 (68.2%) individuals had "healthy mouth," and 131 (31.8%) showed signs of "changing mouth" or "unhealthy mouth."

**TABLE 2:** Overall distribution of OHAT scores in the CI and non‐CI group

Category	CI (*N* = 412) *N* (%)	Non‐CI (*N* = 245) *N* (%)	ALL *N* (%)
0–3 Healthy mouth^a^	281 (68.2)	199 (81.2)	480 (73.1)
4–8 Changing mouthb^b^	122 (29.6)	43 (17.6)	165 (25.1)
>9 Unhealthy mouthc^c^	9 (2.2)	3 (1.2)	12 (0.8)
*p*‐Value	.0013		

^a^ Healthy mouth: maintained by usual care only.

^b^ Changing mouth: observation and monitoring are required.

^c^ Unhealthy mouth: care required and professional dental appointments should be arranged.

On all eight OHAT dimensions, the two groups showed significant differences, with the CI group exhibiting poorer oral health, especially regarding to tongue (*p* = .0007), saliva (*p* = .0011), natural teeth (*p* = .0155) and oral cleanliness (*p* < .001), the proportions scored as "changing mouth" and "unhealthy mouth" were 22.8%, 23.8%, 64.8% and 53.9% in the CI group and 13.0%, 13.5%, 55.8% and 41.6% in the non‐CI group, respectively. The worst dimension was natural teeth, in CI group, 24.3% had over 4 decayed or broken teeth/roots, or less than 4 teeth. The second worsen dimension was oral cleanliness, 10.7% residents with CI showed food particles/tartar/plaque in most areas of the mouth or severe halitosis (Table 3).

**TABLE 3:** Comparison of distribution of scores of OHAT between CI and non‐CI group

Category	CI (*N* = 412)			Mean ± *SD*	Non‐CI (*N* = 245)			Mean ± *SD*	*p*
	0 Healthy	1 Changes	2 Unhealthy		0 Healthy	1 Changes	2 Unhealthy		
Lips	316 (76.7)	92 (22.3)	4 (1.0)	0.24 ± 0.45	204 (83.3)	39 (15.9)	2 (0.8)	0.18 ± 0.40	.1259
Tongue	318 (77.2)	92 (22.3)	2 (0.5)	0.23 ± 0.43	213 (86.9)	28 (11.4)	4 (1.6)	0.15 ± 0.40	**.0007**
Gums and tissues	324 (78.6)	86 (20.9)	2 (0.5)	0.22 ± 0.43	205 (83.7)	39 (15.9)	1 (0.4)	0.17 ± 0.38	.2815
Saliva	314 (76.2)	93 (22.6)	5 (1.2)	0.25 ± 0.46	212 (86.5)	33 (13.5)	0 (0.0)	0.13 ± 0.34	**.0011**
Natural teeth	145 (35.2)	167 (40.5)	100 (24.3)	0.89 ± 0.76	101 (41.2)	107 (43.7)	37 (15.1)	0.74 ± 0.71	**.0155**
Dentures	214 (87.3)	21 (8.6)	10 (4.1)	0.15 ± 0.43	365 (88.6)	34 (8.3)	13 (3.2)	0.17 ± 0.47	.8126
Oral cleanliness	190 (46.1)	178 (43.2)	44 (10.7)	0.65 ± 0.67	143 (58.4)	97 (39.6)	5 (2.0)	0.44 ± 0.54	**<.001**
Pain	349 (84.7)	58 (14.1)	5 (1.2)	0.17 ± 0.40	214 (87.3)	25 (10.2)	6 (2.4)	0.15 ± 0.42	.1872
Total score	2.80 ± 2.36				2.12 ± 2.01				**.0004**

Note

Distribution of scores of per item are expressed as *N* (%); total score is expressed mean ± *SD*. the score of each item is from 0‐3, the oral health condition is from healthy to unhealthy.

Concerning on the influences of oral conditions on OHAT, the CI group showed significantly worsen in oral plaque index (*p* < .001), oral odour (*p* < .001), chewing function (*p* = .0151) and swallowing function (*p* = .0405) than the non‐CI group. In CI group, 58.7% of residents were in the "healthy mouth", with no dental plaque; however, in "unhealthy mouth," 77.7% of residents were with plaque; of these, 33.3% had dental plaque. Similarly, as oral health transitions into the unhealthy category, the severity of oral odour increases. In seniors with "healthy mouth," 56.2% had no oral odour, whereas among those with "unhealthy mouth," 100% exhibited oral odour and 25% had severe oral odour (Table 4).

**TABLE 4:** Influences of oral conditions on scores of OHAT between CI and non‐CI group

Category	CI (*N* = 412)			P for trend	Non‐CI (*N* = 243)			P for trend	P for Homogeneity test
	0–3 Healthy mouth	4–8 Changing mouth	>9 Unhealthy mouth		0–3 Healthy mouth	4–8 Changing mouth	>9 Unhealthy mouth		
Oral environments									
Plaque index^b^ [*N*(%)]				<0.001				0.0112	<0.0001
No plaque	165 (58.7)	39 (32.0)	2 (22.2)		119 (59.8)	17 (39.5)	2 (66.7)		
Nonvisible/ with probe	81 (28.8)	32 (26.2)	4 (44.4)		54 (27.1)	12 (27.9)	1 (33.3)		
Moderate plaque	29 (10.3)	37 (30.3)	0 (0.0)		25 (12.6)	13 (30.2)	0 (0.0)		
Amount plaque	6 (2.1)	14 (11.5)	3 (33.3)		1 (0.5)	1 (2.3)	0 (0.0)		
Oral odour [*N* (%)]				<0.001				–	–
No oral odour	158 (56.2)	36 (29.5)	0 (0.0)		140 (70.4)	24 (55.8)	1 (33.3)		
Suspicious oral odour	70 (24.9)	40 (32.8)	0 (0.0)		38 (19.1)	13 (30.2)	0 (0.0)		
Mild oral odour	43 (15.3)	33 (27.0)	1 (25.0)		21 (10.6)	6 (14.0)	0 (0.0)		
Moderate oral odour	8 (2.8)	9 (7.4)	2 (50.0)		0 (0.0)	0 (0.0)	2 (66.7)		
Severe oral odour	1 (0.4)	3 (2.5)	1 (25.0)		0 (0.0)	0 (0.0)	0 (0.0)		
Serious oral odour	0 (0.0)	1 (0.8)	0 (0.0)		0 (0.0)	0 (0.0)	0 (0.0)		
Oral function									
Chewing function [*N* (%)]				0.0044				0.0403	0.0010
Normal	191 (68.0)	69 (56.6)	3 (33.3)		186 (93.5)	36 (83.7)	2 (66.7)		
Partial chewing disorder	48 (17.1)	31 (25.4)	1 (11.1)		11 (5.5)	7 (16.3)	1 (33.3)		
Total chewing disorder	42 (14.9)	22 (18.0)	5 (55.6)		2 (1.0)	0 (0.0)	0 (0.0)		
Swallowing function [*N* (%)]			0.0066				0.7527	0.0064	
Normal	213 (75.8)	83 (68.0)	3 (33.3)		193 (97.0)	40 (93.0)	3 (100.0)		
Choking cough	14 (5.0)	10 (8.2)	0 (0.0)		2 (1.0)	1 (2.3)	0 (0.0)		
Mild disorder	12 (4.3)	8 (6.6)	1 (11.1)		1 (0.5)	2 (4.7)	0 (0.0)		
Moderate disorder	11 (3.9)	3 (2.5)	0 (0.0)		1 (0.5)	0 (0.0)	0 (0.0)		
Severe disorder	31 (11.0)	18 (14.8)	5 (55.6)		2 (1.0)	0 (0.0)	0 (0.0)		

^a^ Nonvisible plaque: could been measured with probe; moderate plaque: covers more than half of the tooth surface; amount plaque: covers more than two‐thirds of tooth surface;

Focusing on the observed oral health behaviours, only 65 (9.9%) aged residents received oral health assessments, of whom 48 (11.7%) in CI group and 17 (6.9%) in non‐CI group. In CI group, only 153 (37.14%) received oral care procedure, 123 (29.9%) of whom were provided with care by nurses, 30 (7.3%) by nurse assistants. With regarding to the frequency of oral care, 79 (19.2%) received such care daily and 74 (17.9%) more than twice daily. Of those in the non‐CI group, 228 (93.1%) had brushing behaviours and 87.8% used mouthwash. 47.35% brushed more than twice a day and 41.22% brushed before sleep.

### 3.3 Multivariate analysis of the factors associated with OHAT

A multivariate logistic regression analysis was performed to explore the potential influence factors of oral health status. We found that residents in GCFs who receiving oral care had better oral health status (With oral care/without oral care, OR is 0.63, 95CI% (0.43–0.94)). Denture use was a risk factor, those who wearing dentures had worse oral health (No denture use/dentures‐use, OR is 1.95, 95CI% (1.34–2.86)). However, CI was a protective factor in the initial regression analysis. Then a stratified analysis was implemented and the results showed that (Table 5) in CI group, receiving oral care/not receiving oral care, OR is 0.60, 95CI% (0.39–0.92), no dentures/dentures, OR is 2.09, 95CI% (1.31–3.32). Meanwhile, the effects were disappeared in the non‐CI group.

**TABLE 5:** The factors associations with OHAT in multivariate analysis stratified by CI status

Category	CI (*N* = 412)		Non‐CI (*N* = 245)	
	OR	95CI%	OR	95CI%
Providing oral care vs. no oral care	0.60	0.39–0.92	0.87	0.28–2.69
No denture use vs. Denture use	2.09	1.31–3.32	1.71	0.88–3.31

## 4 DISCUSSION

According to our study, oral health status was not desired overall and the situation was even worse among individuals with CI. Oral health status was positively correlated with oral environment and oral function and the CI group showed a poor situation. Furthermore, oral health behaviours were inadequate and most GCFs failed to meet the requirements of the standards of oral care. This study revealed the poor oral health status in GCFs and highlighted the impact of CI on their high levels of oral diseases. These results could be used as a benchmark for subsequent oral studies in this area and could be used for follow‐up oral health improvement programmes to guide future efforts in nursing quality improvement.

In this study, institutional elderly oral health was generally not optimistic and was worsened with CI. Oral problems already occurred among 31% residents with CI, who required medical interventions immediately. Natural teeth appear to be the most problematic domain, as tooth loss, tooth decay or root damage was more prevalent, which was consistent with previous reports that elderly patients with CI were more likely to have fewer remaining natural teeth (Cocco et al., 2018). We also found oral cleanliness as a worsen dimension especially in CI group, while non‐communicable diseases are likely to increase physical inability and self‐care dependence as showed in previous studies (Chen et al., 2019; Wu et al., 2016). In dental pain, CI groups have no difference with cognitively healthy residents, perhaps they could not express themselves properly and existing abnormal behaviours (yelling/screaming; facial convulsion) influenced nurses’ assessment. The CI group showed poorer care than the non‐CI group in gums and tissues and saliva categories, indicating that nurses should evaluate gum tissue and saliva due to the use of long‐term psychotropic drugs, which may lead to reduced saliva secretion with accompanying symptoms like dry mouth, gum swelling and discomfort (Janssens et al., 2017).

Poorer oral condition is associated with diminished status of oral functions, in CI group, among the residents with "healthy mouth," 68% retained normal chewing function and 75.5% had normal swallowing function. However, 55.6% of those in the "unhealthy mouth" group experienced total chewing loss and 55.6% had severe swallowing disorder. Similar as some studies highlighted the importance of oral health as a predictor of oral functions and frailty in older age (Hakeem et al., 2019). On the other hand, chewing and swallowing dysfunction, which results in more food residue left in the mouth, affects the oral environment, the appearance of plaque and oral odour and hence accelerates the deterioration of oral health and even leads to malnutrition.

According to previous studies, increased oral health behaviour helps to improve oral health. However, oral health behaviour is relatively insufficient in GCFs. In our investigation, fewer than 10% of elderly residents received any oral health assessments and 90% were not concerned about oral problems. Even a basic twice‐a‐day tooth‐brushing habit seemed difficult to be adopted, companied by the situation that less than 50% (47.35%) of patients completed the tooth cleaning twice activities and the quality of brushing varied, which was the same case in other researches (Hopcraft et al., 2012a). For those requiring oral care, GCFs have also failed to provide oral care that meets requirements. Our study shows providing oral care is a protective factor in oral health. However, there was only 26.3% of the residents received oral care in GCFs. Interviews with nursing staff revealed difficulties implementing cleaning activities. It was also common for them to encounter sudden resistance, which could interrupt the caring process. Nurses were unwilling to provide oral care due to the fear of managing uncooperative individuals, worrying about potential injures and lack of skills (McNally et al., 2012; Rozas et al., 2017). Another reason was time constraints, as each nurse needed to care for 7.69 elderly people at once, confronting large nursing workload and requiring double time for residents with dementia or CI. Furthermore, oral care appliances are not convenient. An oral care package contains cotton balls and each cotton scrub could wipe away plague and stains but not thorough enough and technically difficult.

The findings of this study have implications for nursing practice, especially for the clinical nurses or nursing administrators working in GCFs around the world. Periodic assessment, observation of oral health conditions of aged residents, must be emphasized through standard policies. And there is a wide gap in knowledge regarding effective strategies specifically to improve oral health in residents with CI, proper oral hygiene education programmes and CI‐focused behaviour management should be developed.

The limitations of this study are related to the recruitment of residents only in Shanghai, which may limit the generalizability of the results. In the process of data collection, cognitive evaluation and oral assessment were conducted by the same nurse, thereby resulting in a possible evaluation bias. Furthermore, the cross‐sectional nature of this study prevented examination of causality between oral health status and cognition impairment. We are planning future longitudinal follow‐up studies with a larger sample size to confirm our findings.

## 5 CONCLUSIONS

Oral health status among aged residents in GCFs was worse among individuals with CI. In the future, oral‐related quality indicators should be part of the process of accreditation in the quality of care in GCFs and systematic oral health surveillance is necessary, especially in CI population.

## 6 RELEVANCE TO CLINICAL PRACTICE

Aged residents in GCFs normally have more oral problems. Periodic and proper oral health evaluations are urgent. Nurses should have the awareness to focus on oral assessment and appropriate intervention.

## CONFLICT OF INTEREST

All authors declare there are no conflicts of interest.

## AUTHOR CONTRIBUTION

Zhang Lingjuan and Gu Liyan designed the study; Chen Wenyao performed the experiments. Li Xianchen contributed to the data analysis; Chen Lan and Gu Liyan contributed to the writing of the paper.

## Data Availability

All data generated or analysed during this study are included in this article.
